# Modulation of Bleomycin-Induced Lung Fibrosis by Pegylated Hyaluronidase and Dopamine Receptor Antagonist in Mice

**DOI:** 10.1371/journal.pone.0125065

**Published:** 2015-04-30

**Authors:** Evgenii Germanovich Skurikhin, Olga Victorovna Pershina, Alena Mikhaylovna Reztsova, Natalia Nikolaevna Ermakova, Ekaterina Sergeevna Khmelevskaya, Vycheslav Andreevich Krupin, Inna Ernestovna Stepanova, Andrew Vladimirovich Artamonov, Andrew Alexandrovich Bekarev, Pavel Gennadjevich Madonov, Alexander Mikhaylovich Dygai

**Affiliations:** 1 Department of Pathophysiology and Regenerative Medicine, Research Institute of Pharmacology and Regenerative Medicine named after E.D. Goldberg, Tomsk, Russia; 2 Limited Liability Company «Scientific Future Management», Novosibirsk, Russia; Children's Hospital Los Angeles, UNITED STATES

## Abstract

Hyaluronidases are groups of enzymes that degrade hyaluronic acid (HA). To stop enzymatic hydrolysis we modified testicular hyaluronidase (HYAL) by activated polyethylene oxide with the help of electron-beam synthesis. As a result we received pegylated hyaluronidase (pegHYAL). Spiperone is a selective D_2_ dopamine receptor antagonist. It was demonstrated on the model of a single bleomycin damage of alveolar epithelium that during the inflammatory phase monotherapy by pegHYAL or spiperone reduced the populations of hematopoietic stem /progenitor cells in the lung parenchyma. PegHYAL also reduced the levels of transforming growth factor (TGF)-β, interleukin (IL)-1β, tumor necrosis factor (TNF)-α in the serum and lungs, while spiperone reduced the level of the serum IL-1β. Polytherapy by spiperone and pegHYAL caused the increase of the quantity of hematopoietic stem/ progenitor cells in the lungs. Such an influx of blood cell precursors was observed on the background of considerable fall level of TGF-β and the increase level of TNF-α in the serum and lungs. These results show pegHYAL reduced the bleomycin-induced fibrosis reaction (production and accumulation of collagen) in the lung parenchyma. This effect was observed at a single and repetitive bleomycin damage of alveolar epithelium, the antifibrotic activity of pegHYAL surpassing the activity of testicular HYAL. The antifibrotic effect of pegHYAL is enhanced by an additional instillation of spiperone. Therapy by pegHYAL causes the flow of CD31^‒^CD34^‒^CD45^‒^CD44^+^CD73^+^CD90^+^CD106^+^-cells into the fibrous lungs. These cells are incapable of differentiating into fibroblast cells. Spiperone instillation separately or together with pegHYAL reduced the MSC-like cells considerably. These data enable us to assume, that pegHYAL is a new and promising instrument both for preventive and therapy of toxic pneumofibrosis. The blockage of D_2_ dopamine receptors with the following change of hyaluronan matrix can be considered as a new strategy in treatment of pneumofibrosis.

## Introduction

Pneumofibrosis includes a heterogeneous group of lung disorders, characterized by a progressing and irreversible destruction of alveolar epithelium architecture, caused by scar-forming, which leads to the failure of gases interchange, and, finally, to death from respiratory failure [1]. Idiopathic pulmonary fibrosis (IPF) is the most serious form of pneumofibrosis with unknown etiology. Smoking and environmental problems are potential risk factors for IPF (coal-, stone-, wood-, metal- dust, sand silicon dioxide) [2–9]. IPF of lungs can grow as a complication after virus infections, radiotherapy and chemical therapy, after adverse effects of environmental spray toxins [10–13]. With no symptoms IPF lasts for a long time. However, later it inevitably leads to disability of the patient. The average life expectancy after the diagnosis of pneumofibrosis has been made is 2–3 years; according to other data not more than 6 years [14–16]. The existing set of treatment procedures for IPF is limited and ineffective. The clinical practice is focused mainly on treatment of complications and supporting therapy [16, 17].

Hyaluronidases are a group of enzymes that regulate hyaluronic acid metabolism and remodel the extracellular matrix [18]. With bleomycin-induced pneumofibrosis the testicular HYAL inhibits the destruction of alveolar epithelium cells and the growth of pulmonary fibrosis in mice [19]. However, owing to enzymatic hydrolysis the concentration of HYAL in lungs decreases fast. As a result, the progress of pneumofibrosis is observed. Covalent attachment of the vehicle to the pharmalogically active molecule of interest has become a traditional formula of prodrug creation [20, 21]. During pegylation polyethylene oxide is the most often used polymer [22, 23]. The clinical effects of pegylated biologically active peptides surpass the corresponding characteristics of unmodified molecules. The pharmacological potential of pegylated drugs improves on account of rise of solubility in water, resistance to enzyme activity, the address delivery of the drug to the target [22, 23]. According to our hypothesis, the preservation of the structure and pharmacological activity of HYAL in aggressive medium is possible with the help of pegylation.

The question of rise in effectiveness of medical treatment is fundamental for the clinic. Dopamine and dopamine receptors (D_1–4_ subtypes) are defined in lung tissue [24]. Dopamine receptors of D_1_ and D_2_ subtypes in lung vessels are described [25, 26]. Proceeding from these results, the existence of dopamine mechanisms of lung diseases is not excluded. In our opinion, the use of antagonists of dopamine receptors can change pneumofibrosis in monotherapy and in assignment with preparations, changing the profibrotic matrix of hyaluronan.

Mesenchymal stem cells (MSC) and hematopoietic stem cells (HSC) are multipotent cells, which can differentiate into cells of stromal and hematopoietic lineage correspondingly. Therapy on the basis of endogenous stem cells (SC) is considered as a new promising approach in chronic lung diseases. However, cell therapy using endogenous SCs has obstacles, namely: the participation of some fraction in pathogenesis of diseases is not clear, also if SCs change with the prescription of medicine.

In the light of this, we tried to study the action of pegylated HYAL on the development of bleomycin-induced pneumofibrosis, the effects of pegHYAL being compared with the effects of testicular HYAL. We studied the effectiveness of pneumofibrosis treatment, using the polytherapy of spiperone (selective D_2_ dopamine receptor antagonist) and pegHYAL. Besides, the effect of pegHYAL and spiperone on inflammatory cytokines, hematopoietic stem/progenitor cells, and MSC-like cells was studied.

These investigations demonstrated for the first time, that intranasal administration of pegHYAL decreases the bleomycin-induced fibrous reaction in lung parenchyma. A certain effect is observed when the preparation is assigned during the phase of inflammation and fibrotic phase with a single or repetitive bleomycin damage of alveolar epithelium. The antifibrotic activity of pegHYAL surpasses the one of testicular HYAL. We suggest that the antifibrotic effect of pegHYAL be intensified with an additional intraperitoneal instillation of spiperone. Besides, pegHYAL and spiperone affect TGF-β, IL-1β, TNF-α, hematopoietic stem / progenitor cells and MSC-like cells. These new results show that the blockage of D_2_ dopamine receptors with the following change of hyaluronan matrix should be considered as a new strategy in pneumofibrosis therapy.

## Materials and Methods

### Animals

Male C57BL/6 mice (8–10 weeks old) were purchased from the Dept. of Biomodels, Research Institute of Pharmacology and Regenerative Medicine named after E.D. Goldberg (Veterinary Certificate number 0007293), housed under pathogen-free conditions with food and water ad libitum. All animal care and experimental procedures were approved by Care Committee of the Research Institute of Pharmacology and Regenerative Medicine (Protocol No 71052014). During the study 340 mice were used.

### Reagents

Bleomycin (BLM) sulphate was purchased from Bristol Myers-Squibb (Blenoxane; São Paulo, Brazil). Bull testicular hyaluronidase—HYAL (kindly provided for research by LLC «Scientific Future Management», Novosibirsk, Russia). Pegylated hyaluronidase (pegHYAL) is a HYAL which modified by activated polyethylene oxide of molecular weight 1500 Da using electron-beam synthesis (kindly provided for research by LLC «Scientific Future Management», Novosibirsk, Russia). Spiperone is a selective D_2_ dopamine receptor antagonist, α_1B_-adrenoceptor antagonist; mixed 5-HT_2A_/5-HT_1_ serotonin receptor antagonist (Sigma, USA).

### HYAL, PegHYAL and Spiperone experimental design and bleomycin lung injury

Control animals were administered a single intratracheal 0.03 ml 0.9% NaCl.

Alveolar epithelial injury in C57BL/6 mice induced by a single intratracheal bleomycin administration at a dose 80 μg/mouse in 0.03 ml of 0.9% NaCl, which was slowly instilled in the tracheal lumen [19, 27]. Thus, partially reversible pulmonary fibrosis was modeled. These animals formed the BLM control. In model studies of partially reversible pulmonary fibrosis intratracheal administration of BLM was taken as the 0 day of the experiment.

Mice were injected intratracheally by single dose of BLM at dose 80 μg/mouse in 0.03 ml of 0.9% NaCl for modeling of repetitive toxic injuries of alveolar epithelium. The first intratracheal BLM administration was taken as the 0 day of the experiment. BLM was administered intranasally at a dose 80 μg/mouse in 0.015 ml of 0.9% NaCl on the 7, 14, 21, 28-th day of experiment. Thus irreversible pulmonary fibrosis was simulated. These animals formed the BLM control.

All procedures were performed under anesthesia induced by intraperitoneal injection of chloral hydrate (400 mg kg^-1^ intraperitoneal). At the designated time points (days 7, 14, 21, 40 or 60 after BLM administration) mice were humanely sacrificed by CO_2_ asphyxiation, both lungs were removed and frozen immediately in liquid nitrogen. Tissue samples were stored at −80°C until further processing.

#### Administration of drugs and groups of animals on the model of partially reversible pulmonary fibrosis

In the conditions of partially reversible pulmonary fibrosis pegHYAL (8 U/18 μ buffer/mouse) and HYAL (8 U/18 μ buffer/mouse) was administered intranasally on the 1, 3, 7, 10, 13th day of the experiment. Spiperone dose of 1.5 mg/kg in 0.1 ml 0.9% NaCl was injected intraperitoneally 3 hours since and on the 1st, 3rd, 7th, 10th, 13th days after BLM instillation. Submitted mode of administered drugs is preventive. When studying the effects of complex prevention of partially reversible pulmonary fibrosis progression pegHYAL (8 U/18 μ buffer/mouse) was administered intranasally on the 1, 3, 7, 10, 13-th day of the experiment, in 1 hour after administration of pegHYAL spiperone was administered intraperitoneally (1.5 mg/kg/ 0.1 ml 0.9% NaCl). Control groups were consisted of intact mice.

Mice with partially reversible pulmonary fibrosis were in the pathological control group. Mice with partially reversible pulmonary fibrosis and drugs made experimental groups: experimental group 1—prevention by 0.9% NaCl; experimental group 2—HYAL prevention; experimental group 3—pegHYAL prevention; experimental group 4—spiperone prevention; experimental group 5—prevention by consistently administered pegHYAL and spiperone.

#### Drugs administration on the model of irreversible pulmonary fibrosis

Under conditions of irreversible pulmonary fibrosis pegHYAL (8 U/18 μ buffer / mouse) and HYAL (8 U/18 μ buffer / mouse) was administered intranasally on the 10–16, 18–23, 25–30, 36–42th day of the experiment. Spiperone was injected intraperitoneally in dose 1.5 mg/kg in 0.1 ml of 0.9% NaCl on the 10–16, 18–23, 25–30, and 36–42th day of the experiment. Presented mode of drugs administration is therapeutic. Studying the effects of complex treatment of irreversible pulmonary fibrosis pegHYAL (8 U/18 μ buffer / mouse) was administered intranasally on the 10–16, 18–23, 25–30, 36–42th day of the experiment, in 1 hour after pegylated enzyme instillation spiperone was administered intraperitoneally (1.5 mg/kg / 0.1 ml 0.9% NaCl).

Control group was consisted of intact mice. Mice with irreversible fibrosis were in the pathological control group. Mice with irreversible pulmonary fibrosis and drugs were in experimental groups: experimental group 7—treatment by 0.9% NaCl, experimental group 8—treatment by HYAL, experimental group 9—treatment by pegHYAL, experimental group 10—treatment by spiperone, experimental group 11—treatment by consistently administered pegHYAL and spiperone.

### Morphological examination of blood and bone marrow

#### Blood

Leucocyte, granulocyte and lymphocyte counts were obtained by classical methods as was described earlier [28]. Hemogram was counted for 100 cells and after that absolute blood concentration of granulocytes and lymphocytes was calculated.

#### Bone marrow

To study bone marrow cellularity murine femurs were isolated, separated from surrounding tissue and the central channels were rinsed with 1 ml of 3% acetic acid solution. The cells from bone marrow were obtained by classical methods as was described earlier [28, 29]. 400 cells were counted for myelogram and the absolute amount of immature and mature neutrophilic granulocytes, lymphocytes and normoblasts in bone marrow was calculated.

### Histopathological studies of lung

For histological research lung right lobe was collected and immediately fixed in 10% formalin. Specimens were processed, embedded in paraffin and cut into four to six lm sections and were stained by hematoxylin-eosin (H&E) to assess lung architecture and inflammatory cells. To count collagen fibers in lung parenchyma the histological slides were stained by picrofuchsin using Van Gieson method [30, 31]. At least 10 photomicrographs without overlapping across the cut surface of the lung tissue at 100 x magnification were taken for each experimental animal Used system consists of a microscope (Axio Lab.A1, Carl Zeiss MicroImaging GmbH; Göttingen, Germany) with a video camera (AxioCam ERc5s, Carl Zeiss; Göttingen, Germany), connected to a personal computer. Gathered images were processed using the software AxioVision Rel.4.8.2. The content of collagen fibers in lung was determined using a function for counting the area of the object in the image. Broncho-vascular strands were carefully removed from the analyzed areas.

### Flow Cytometric Analysis

Membrane receptors expression of mesenchymal stem cells in lungs was analyzed using BD surface markers (BD Biosciences, USA).The mononuclear cells were stained for 30 minutes with the following antibodies: anti-rat CD90 (Thy-1)/mouse CD90.1 (PerCP), CD34 FITS, CD45 (APC-Cy7), CD73 (PE), CD106 (VCAM-1) FITS, CD44 (Pgp-1, Ly-24) APC and anti-mouse CD31 (PECAM-1) APC (BD Biosciences, USA). Also the following control groups of isotype: FITS Rat IgG2a, PerCP Mouse IgG1, APC Rat IgG2b, APC-Cy7 Rat IgG2b, PE Rat IgG2a were used. The labeled cells were thoroughly washed with PBS × 2 and analyzed on FACSCanto II (Becton Dickinson) with the with FACS Diva software program. A minimum of 100,000 events were recorded for each tube.

Membrane receptor's expression of murine HSCs from lung were assayed according to the protocol for BD Mouse Hematopoietic Stem and Progenitor Cell Isolation Kit (BD Biosciences, US). The labeled cells were thoroughly washed with PBS × 2 and analyzed on FACSCanto II (Becton Dickinson) with the with FACS Diva software program. A minimum of 100,000 events were recorded for each tube.

### Culture studies

Multilineage differentiation and characterization of lung MSC to adipocyte, chondrocytes and osteoblast lineages was previously performed and was described earlier by us [28, 29]. The expression of CD45 receptor on lung cells was previously investigated, after that cells suspension was separated into adherent and non-adherent fractions.

#### Cloning of the fibroblast colony forming units (CFU- F)

Cultures were performed in 24-well plates at 1 × 10^5^ by adherent cells of lung/1 mL of the base culture medium based on the D-MEM with 10% FBS (HyClone, USA), 280 mg/L L-glutamine, 50 mg/L gentamicin, 25 ng/mL fibroblast growth factor (FGF-basic), 30% methylcellulose solution (all supplements from Sigma, USA). Incubation is performed at 37°C and 5% CO_2_ atmosphere with an absolute humidity for 10 days. At the end of the study CFU-F (> = 50 cells per colony) are counted using an inverting microscope and morphological analysis of colonies is carried out [28].

### ELISA Assay

Hyaluronic acid levels were determined by ELISA according to the manufacturer instructions (Cusabio Biotech CO., Ltd, China). Right lobes of the lung were isolated, weighed, and frozen immediately. Sensitivity was > 15.6 pg/ml for hyaluronic acid.

#### Hydroxyproline and collagen type I measurements

Hydroxyproline and collagen type I were quantified in homogenate of right lung lobes. Hydroxyproline and collagen type I were determined by ELISA according to manufacturer instructions (Cusabio Biotech CO., Ltd, China). The right lung lobes were excised and snap frozen after having measured the wet weight. Sensitivities were> 1.95 ng/ml for hydroxyproline and> 0.039 ng / ml for collagen type I.

#### Total soluble collagen assay

The right lung lobes homogenate supernatants were placed in 1.5 mL tubes. Sircol-dye was added, the content of the tubes homogenized for 30 min and centrifuged for 10 min (10,000 × g).The pellets were dissolved with alkaline reagent. Absorbance was read at 540 nm. The total soluble collagen was determined using a standard curve for the Sircol^TM^ assay (Biocolor Ltd, UK) according to manufacturer's instructions Results were expressed as mg collagen per right lung.

#### Cytokines measurements

The concentrations of IL-1β, TNF-α and TGF-β in lung parenchyma and serum were determined by ELISA according to manufacturer instructions (BD Biosciences). Sensitivities were >10 pg/mL for IL-1β and TNF-α and >10 ng/mL for TGF-β.

### Statistical analysis

Data are expressed as mean ± standard error of mean. Statistical variations were determined by analysis of variance (ANOVA) and Student's t-test. Values of P < 0.05 were considered significant.

## Results

### Morphological study of the lung right lobe

**Fig 1:**
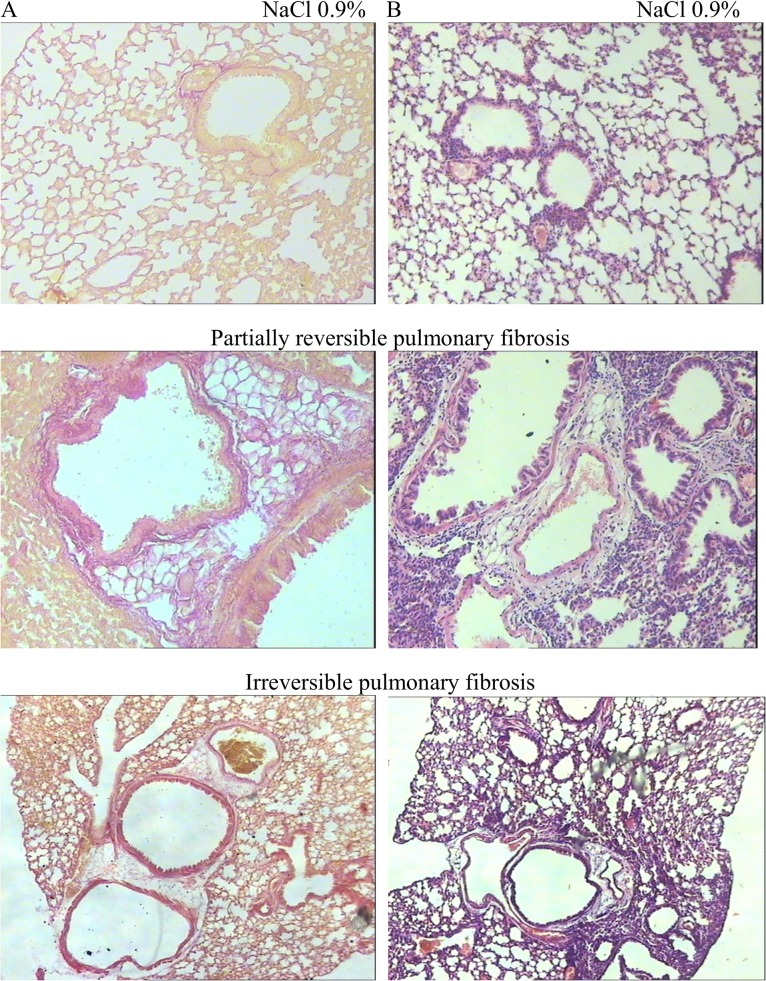
Effect of bleomycin on the lung architecture in C57Bl/6 mice. Comparison of the lung architecture in C57Bl/6 mice after BLM instillation (21st day of the experiment—partially reversible pulmonary fibrosis or 60th day of the experiment—irreversible pulmonary fibrosis) or 0.9% NaCl (sham treatment), as shown by hematoxylin-eosin (**A**) and picrofuchsin (**B**) staining of representative tissue sections. The photomicrographs were taken using an Axio Lab.A1 (Carl Zeiss MicroImaging GmbH; Göttingen, Germany) microscope and AxioCam ERc5s digital camera. All photomicrographs were at 100 × magnification.

In histopathological examinations in comparative aspect we evaluated the influence of single and repeated bleomycin injury of the alveolar epithelium (Fig 1). With a single dosing of cytostatic pulmonary tissue injury in C57BL/6 mice proceeds step by step. In our investigations the highest intensity of inflammatory response was achieved on the 14th day of experiment. The proliferation of connective tissue started from 7th day and was followed to the 60th day (Fig 1). Pulmonary fibrosis pattern was the most evident on the 21st day after bleomycin injection. To the 60th day the inflammatory response intensity in lungs decreased and collagen content reduced over 50%.

#### Partially reversible pulmonary fibrosis

**Fig 2:**
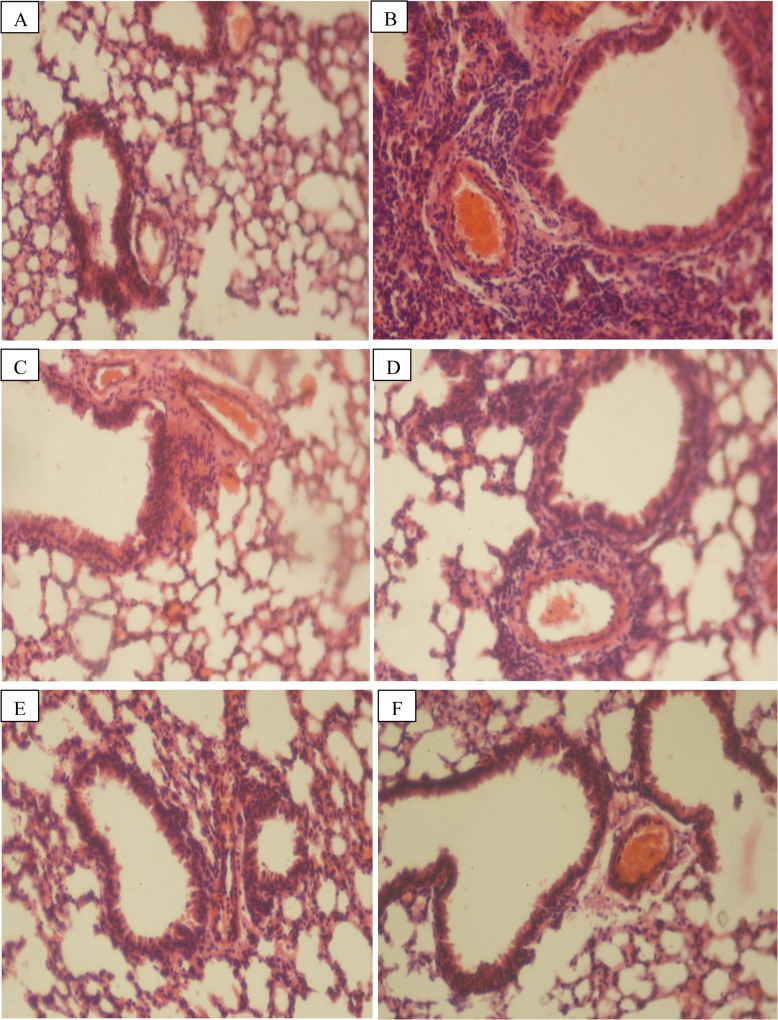
Photomicrographs of representative lung sections obtained from C57BL/6 mice after a single bleomycin instillation (stained by hematoxylin-eosin). Tissues were stained with hematoxylin-eosin to investigate inflammatory cells accumulation (21st day of the experiment). (**A**) Mice receiving intratracheal 0.9% NaCl, (**B**) Mice receiving intratracheal BLM, (**C**) Mice with fibrosis spiperone treated, (**D**) Mice with fibrosis HYAL treated, (**E**) Mice with fibrosis pegHYAL treated, (**F**) Mice with fibrosis pegHYAL and spiperone treated. The photomicrographs were taken using an Axio Lab.A1 (Carl Zeiss MicroImaging GmbH; Göttingen, Germany) microscope and AxioCam ERc5s digital camera. All photomicrographs were at 100 × magnification.

**Fig 3:**
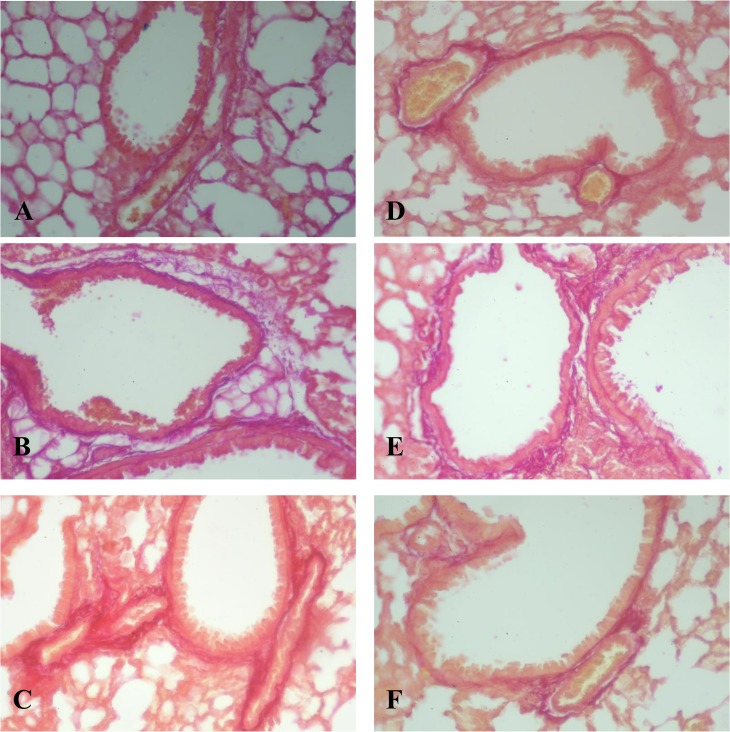
Photomicrographs of representative lung sections obtained from C57BL/6 mice after a single bleomycin instillation (stained by picrofuchsin). Tissues were stained with picrofuchsin to determine the collagen content (21st day of the experiment). (**A**) Mice receiving intratracheal 0.9% NaCl; (**B**) Mice receiving intratracheal BLM; (**C**) Mice with fibrosis spiperone treated; (**D**) Mice with fibrosis HYAL treated; (**E**) Mice with fibrosis pegHYAL treated; (**F**) Mice with fibrosis pegHYAL and spiperone treated. Dark pink stains are collagenous deposits. The most expressed collagen fibers deposition after BLM injection was observed on the 21st day. The photomicrographs were taken using an Axio Lab.A1 (Carl Zeiss MicroImaging GmbH; Göttingen, Germany) microscope and AxioCam ERc5s digital camera. All photomicrographs were at 100 × magnification.

Figs 2 and 3 show the morphological picture of the lung from mice of the control group (NaCl injection), bleomycin control (BLM injection) and the experimental groups (BLM injection and/or HYAL, pegHYAL, spiperone and combination of pegHYAL and spiperone) on the 21st day after BLM instillation. BLM injection was induced infiltration of the alveoli by lymphocytes, neutrophils, plasma cells and macrophages (Fig 2). HYAL and pegHYAL did not induce alterations in the lung architecture, spiperone and combination of pegHYAL and spiperone reduced the degree of alveoli desquamation in alveolar lumen (Fig 2). Staining of lung by Van Gieson's picrofuchsin revealed that at partially reversible fibrosis HYAL reduced matrix deposition (up 66.5%) in the lung of animals with fibrosis compared to untreated animals on the 14th day of the experiment (Table 1, Fig 3). Isolated administered pegHYAL and spiperone reduced matrix deposition on the 7th, 14th, 21st days after BLM instillation. Subsequently administered pegHYAL and spiperone prevented the deposition of fibrous masses in the lungs from mice with fibrosis more effectively compared with sick mice receiving drugs alone. As it is seen from Table 1 on the 7, 14, 21-st day of the experiment the matrix deposition in experimental group 4 didn’t differ from the index in the intact control.

**Table 1:** Effects of hyaluronidase (HYAL) and Spiperone treatment on the content of the connective tissue in the lungs of C57BL/6 mice at the partially reversible pneumofibrosis (% of the area of lung tissue).

Groups	Day after single dose bleomycin instillation		
	7	14	21
**Mice received intratracheal 0.9% NaCl**	1.13±0.07	1.14±0.03	1.15±0.06
**Mice with fibrosis 0.9% NaCl treated**	3.07±0.09*	3.91±0.07*	4.26±0.14*
**Mice with fibrosis HYAL treated**	3.05±0.17*	2.60±0.21* &	3.24±0.07*
**Mice with fibrosis pegHYAL treated**	2.22±0.12* &	2.12±0.12* &	2.24±0.18* &
**Mice with fibrosis Spiperone treated**	2.09±0.15* &	2.75±0.12* &	2.12±0.13* &
**Mice with fibrosis pegHYAL and Spiperone treated**	1.45±0.08 &	1.22±0.14&	1.40±0.09&

The content of collagen fibers in lung was determined using a function for counting the area of the object in the image. Broncho-vascular strands were carefully removed from the analyzed areas. The relative fibrotic tissue area is calculated of by the formula: X=Σax100/(S−Σb) where: a—is the amount of pixels occupied by fibrotic tissue in 10 pictures of one drug, S—is the number of pixels corresponding to the total area of the image (using this camera and software—4,423,680), b—a sum of pixels occupied by the empty part of the glass slide in 10 pictures of one drug.

Data represent mean of 3 independent experiments, n = 10/group. Results are presented as mean and SEM.

*—compared to the mice received intratracheal 0.9% NaCl (P <0.05),

&—compared to the mice received intratracheal BLM and 0.9% NaCl treated (P<0.05) t test was used.

#### Irreversible pulmonary fibrosis

Bleomycin reproduces many of the IPF typical features in humans. Such criteria as intra-alveolar buds, collagen intramural inclusion and alveolar space closing are presented in patients with IPF and in BLM treated animals [32]. Meanwhile, the aspect of slow and irreversible progression of pulmonary fibrosis in experimental animals, administered once by BLM, is irreproducible [33, 34]. In the clinic, patients with IPF are presented at advanced stage of the disease. Most potential antifibrotic compounds do not have the expected effect in clinical trials, or their activity is incomparably lower than in animal models of lung fibrosis [35]. The reason for this state of affairs appears the limitation of preclinical studies of potential antifibrotic compounds by single toxic lung injury models (partially reversible pulmonary fibrosis) and assessment of their effectiveness in prevention mode of administration. Thus, on the first place there is the search of compounds that are effective in terms of pulmonary fibrosis progression. On the model of repeated alveolar epithelial injury by BLM (irreversible fibrosis), we investigated the antifibrotic effects of pegHYAL at the therapeutic mode of administration. Doses and methods of administration are presented in the section "Material and Methods". The survival rate for C57BL/6 mice after BLM instillation presented in the S1 Table.

Administration of HYAL, pegHYAL and spiperone alone significantly reduce the amount of connective tissue in the lungs of animals with irreversible fibrosis compared to untreated animals on the 21st, 40th day of the experiment. As can be seen from Table 2, treatment by pegHYAL and spiperone is more effective compared to the HYAL. After cessation of therapy (60th day of the experiment) the rate in the animals groups with HYAL, pegHYAL and spiperone increased. Treatment of irreversible pulmonary fibrosis of consistently administrated pegHYAL and spiperone is more effective than monotherapy. So, on the 21st, 40th day of the experiment pathological collagen deposition in the lung parenchyma in sick animals is not registered. On the 60th day the content of matrix deposition under the influence of pegHYAL and spiperone significantly decreased to 46.64% compared to untreated irreversible pulmonary fibrosis. At monotherapy by HYAL, pegHYAL and spiperone the index decreased to 98%, 80% and 83%, respectively, as related to untreated irreversible pulmonary fibrosis.

**Table 2:** Effects of hyaluronidase (HYAL) and Spiperone treatment on the content of the connective tissue in the lungs of C57BL/6 mice at the irreversible pneumofibrosis (% of the area of lung tissue).

Groups	Day after bleomycin instillation		
	21	40	60
**Mice received intratracheal 0.9% NaCl**	1.15±0.06	1.12±0.18	1.23±0.16
**Mice with fibrosis 0.9% NaCl treated**	5.13±0.75*	4.12±0.40*	5.96±0.93*
**Mice with fibrosis HYAL treated**	2.84±0.32* &	2.61±0.41*&	5.90±0.79*
**Mice with fibrosis pegHYAL treated**	1.87±0.19* &	2.09±0.19*&	4.78±0.64*
**Mice with fibrosis Spiperone treated**	1.86±0.15* &	1.52±0.13*&	4.96±0.31*
**Mice with fibrosis pegHYAL and Spiperone treated**	1.05±0.09 &	1.22±0.11 &	2.78±0.19*&

The content of collagen fibers in lung was determined using a function for counting the area of the object in the image. Broncho-vascular strands were carefully removed from the analyzed areas. Data represent mean of 3 independent experiments, n = 10/group. Results are presented as mean and SEM.

*—compared to the mice received intratracheal 0.9% NaCl (P <0.05),

&—compared to the mice received intratracheal BLM and 0.9% NaCl treated (P<0.05), t test was used.

These data showed that under the conditions of single and repeated alveolar epithelial injury by BLM, administered alone pegHYAL and spiperone prevent the development of pulmonary fibrosis. Effect of the drugs was observed in the prophylactic and therapeutic mode of administration. The activity of pegHYAL exceeds HYAL. Sequential administration of pegHYAL and spiperone reduces the amount of matrix deposition in lung more effectively than drugs administered alone.

### ELISA assay

#### Partially reversible pulmonary fibrosis

At the model of partially reversible pulmonary fibrosis by ELISA we evaluated levels of cytokines (TGF-β, IL-1β and TNF-α) in the lung parenchyma and serum on the 3rd after BLM treatment (S1 Fig). On 21^st^ day of the experiment by ELISA we evaluated levels type I collagen, hydroxyproline, total collagen and hyaluronic acid in the homogenates of the lung right lobe. Effects of the drugs were studied in the period of inflammation (3^rd^ day of the experiment) and greatest change of histopathological lung indicators in the treatment of partially reversible pulmonary fibrosis on the 21st day of the experiment.

The content of TNF-α and TGF-β1 in bleomycin-injured lung was reduced at day 3 post-BLM administration (S1 Fig). The content of TGF-β1 (up to 5033%) and IL-1β (up to 541%) in serum of mice with fibrosis on 3rd day of experiment was higher than that of serum from healthy mice, however the concentration of TNF-α was reduced (to 38%) compared with healthy mice (S1 Fig). PegHYAL was reduced the concentration of TNF-α (to 1.3%), IL-1β (to 72%), TGF-β1 (to 12.5%) in serum and TGF-β (to 11%) in BLM-injured lung compared to untreated mice with fibrosis (S1 Fig). Spiperone was reduced levels of TNF-α (to 3.6%), IL-1β (to 50.2%) in the serum of infected animals, at the same time, the concentration of these cytokines in the BLM-injured lung homogenates was increased (respectively up to 169% and 149%). The concentration of serum and lung TGF-β after polytherapy with spiperone and pegHYAL significantly was reduced (respectively to 1.3% and up to 34%) in mice with fibrosis compared with mice without treatment (S1 Fig).

Thus, pegHYAL and spiperone separately and together reduced levels of pro-inflammatory cytokines in serum on the 3 day of experiment. The monotherapy of spiperone increased the concentration of TGF-β, IL-1β in the bleomycin-injured lungs. Independent of treatment (monotherapy or polytherapy) pegHYAL reduced TGF-β in bleomycin-injured lungs.

On the 21st day of experiment there is a significant increase in the levels of collagen type I (up to 192%), hydroxyproline (298%), the total collagen (182%), and hyaluronic acid (up to 352%) in animals lung homogenates with partially reversible pulmonary fibrosis related to intact control. HYAL and pegHYAL reduce the concentration of collagen type I (to 83.3 and 80.96%, respectively), hydroxyproline (to 64.96% and 49.38%, respectively) and total collagen (to 78.06% and 65.29%, respectively) in lung with fibrosis compared to untreated animals (Table 3). Additionally pegHYAL reduces the level of HA in lungs with fibrosis to 35.1% relative to untreated animals. Spiperone, administered singularly and consecutively with pegHYAL, reduces high levels of collagen type I (to 67.2% and 70.55%, respectively), hydroxyproline (to 65.3% and 40%, respectively), total collagen (to 79.5% and 58.25%, respectively) and HA (to 43.54% and 32.99%, respectively) in lungs with fibrosis in comparison with untreated animals (Table 3). It should be noted that the subsequently administered pegHYAL and spiperone significantly reduce hydroxyproline concentration and total collagen than monotherapy.

**Table 3:** Effects of hyaluronidase (HYAL) and Spiperone treatment on collagen type I, hydroxyproline, total soluble collagen, hyaluronic acid levels after the single dose intratracheal bleomycin instillation.

Groups	Collagen type I	Hydroxyproline	Total soluble collagen	Hyaluronic acid
**Mice received intratracheal 0.9% NaCl**	121.1±11.7	2915±240	69.6±6.3	28054±1901
**Mice with fibrosis 0.9% NaCl treated**	233.3±22.4*	8714±651*	127.2±11.4*	98948±3358*
**Mice with fibrosis HYAL treated**	194.6±17.3*	5661±402●	99.3±8.1	98611±7754*
**Mice with fibrosis pegHYAL treated**	188.9±15.4	4303±321●	83.05±5.5●	34736±2604●
**Mice with fibrosis Spiperone treated**	156.8±12.1●	5693±298●	101.2±6.1●	43086±2857* ●
**Mice with fibrosis pegHYAL and Spiperone treated**	164.6±11.1●	3486±287●	74.1±7.2●	32649±1921●

Hydroxyproline, collagen type I, total soluble collagen and hyaluronic acid levels were measured in homogenate of right lung lobes from C57BL/6 mice on 21^st^ day after BLM treatment. Hydroxyproline, collagen type I and HA were assayed by ELISA according to manufacturer instructions (Cusabio Biotech CO., Ltd, China). The right lung lobes were excised and snap frozen after having measured the wet weight. Sensitivities were >1.95 ng/mL for hydroxyproline, >0.039 ng/mL for collagen type I and >15.6 pg/mL for HA. The total soluble collagen was determined using a standard curve for the Sircol^TM^ assay (Biocolor Ltd, UK) according to manufacturer’s instructions. Results were expressed as μg collagen per right lung.

Data represent mean of 3 independent experiments, n = 5/group. Results are presented as mean and SEM.

*—compared to the mice received intratracheal 0.9% NaCl (P <0.05),

●—compared to the mice received intratracheal BLM and 0.9% NaCl treated (P<0.05), t test was used.

Thus, in conditions of a single alveolar epithelial injury by BLM, singularly administered pegHYAL and spiperone reduce the level of collagen type I, hydroxyproline concentration, total collagen and HA. Subsequent administration of pegHYAL and spiperone reduces the levels of hydroxyproline and total collagen in bleomycin lungs more effectively than drugs administered alone.

### Immunophenotypic characterization of lung mononuclear cells

#### Hematopoietic stem cells and hematopoietic progenitor cells

**Fig 4:**
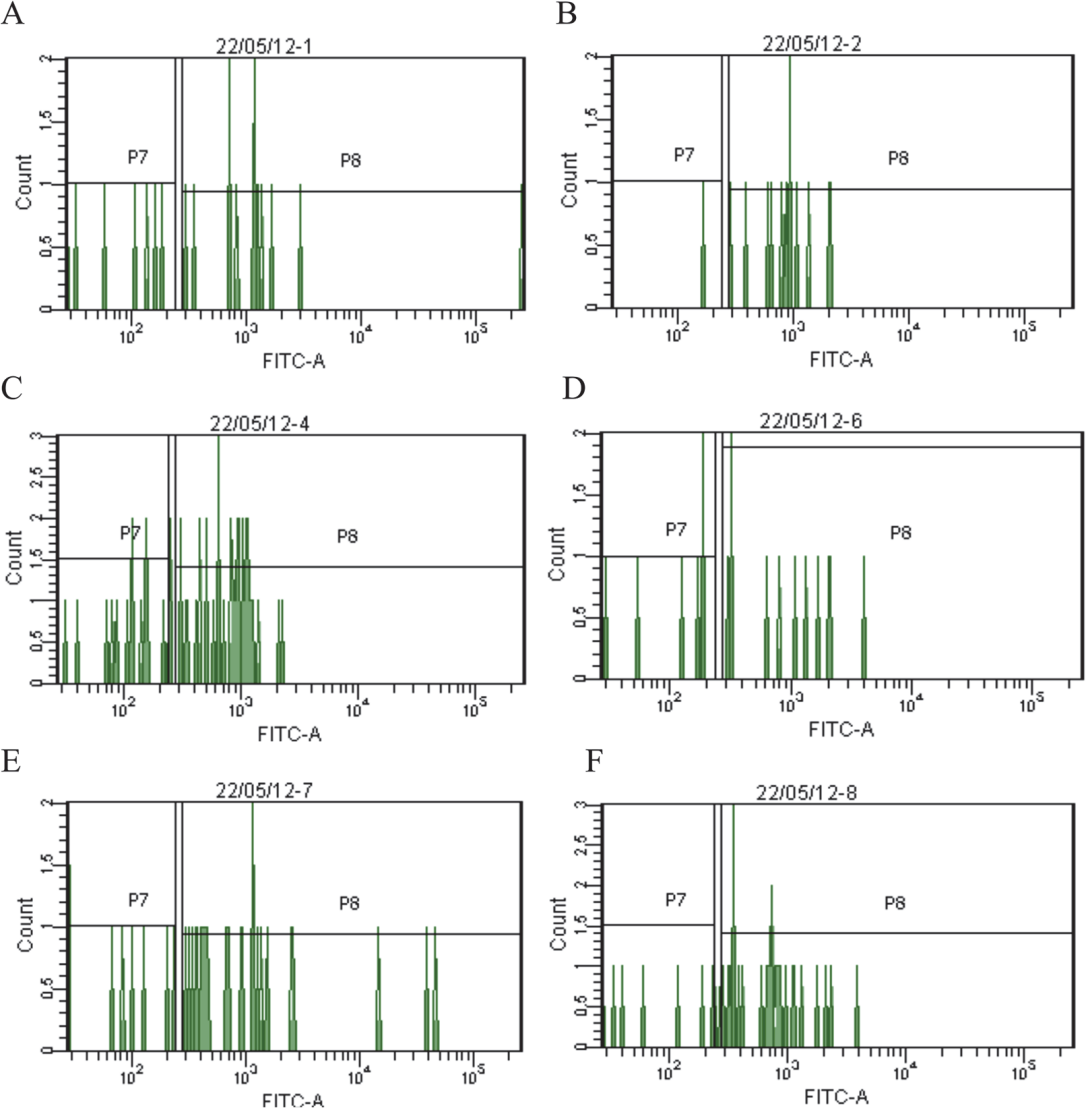
Characterization of hematopoietic stem cells isolated from lung of C57BL/6 mice (7th day of the experiment). The phenotype of cells from lung was studied according to the protocol for hematopoietic stem cells (BD Biosciences). The HSC population taken through a Lin^-^ selection (not shown) and then Sca1^+^ and c-kit^+^ (not shown), is shown gated displayed for CD34^-^ (P7) and CD34^+^ (P8). The Lin^-^Sca1^+^c-kit^+^CD34^-^ cells are LT-HSCs and the Lin^-^Sca1^+^c-kit^+^CD34^+^ cells are considered ST-HSCs. Thus, all two of these populations can be readily sorted from one sample. (**A**) HSCs isolated from mice after intratracheal 0.9% NaCl administration; (**B**) HSCs isolated from mice after intratracheal BLM administration; (**C**) HSCs isolated from mice after intratracheal BLM administration and treated spiperone; (**D**) HSCs isolated from mice after intratracheal BLM administration and treated pegHYAL; (**E**) HSCs isolated from mice after intratracheal BLM administration and treated spiperone and pegHYAL; (**F**) HSCs isolated from mice after intratracheal BLM administration and treated HYAL. Dot plots are representative figures of three independent experiments with the mean from three independent experiments.

On the 7th day of the experiment in the lung parenchyma of mice from intact control with partially reversible pulmonary fibrosis, and in mice with partially reversible pulmonary fibrosis, treated by pegHYAL and spiperone administered alone and sequentially, LT-HSCs (Lin^-^Sca-1^+^c-kit^+^CD34^-^), ST-HSCs (Lin^-^Sca-1^+^c-kit^+^CD34^+^) and hematopoietic progenitor cells (Lin^-^Sca-1^+^c-kit^+^) were studied (Fig 4). The drugs were administered in a preventive mode. We previously investigated functional properties of HSCs derived from lung and described earlier [28, 29].

On the 7th day after a single intratracheal BLM instillation a significant increase of Lin^-^Sca-1^+^c-kit^+^CD34^-^ -cells (up to 171%), Lin^-^Sca-1^+^c-kit^+^CD34^+-^cells (up to 333%) and Lin^-^ Sca-1^+^c-kit^+^-cells (up to 204%) in the lung in relation to the intact control was observed (Table 4). PegHYAL and spiperone reduced the number of LT-HSCs (up to 25% and 80.8%, respectively), ST-HSCs (up to 26.6% and 50% respectively) and hematopoietic progenitor cells (up to 27.7% and 71.1%, respectively) in bleomycin lungs of mice relative to untreated animals with partially reversible pulmonary fibrosis. At sequential administration of pegHYAL and spiperone the number of LT-HSCs decreased to 58.3% as compared to untreated animals with partially reversible pulmonary fibrosis, ST-HSCs up to 66.6%, the hematopoietic progenitor cells up to 88.8%.

**Table 4:** Effects of pegylated hyaluronidase (pegHYAL) and spiperone treatment on HSCs and hematopoietic progenitor cells.

Groups	Lin^-^Sca-1^+^c-kit^+^ CD34^-^ -cells	Lin^-^Sca-1^+^c-kit^+^ CD34^+^-cells	Lin^-^Sca-1^+^c-kit^+-^cells
**Mice received intratracheal 0.9% NaCl**	0.07±0.005	0.018±0.001	0.088±0.007
**Mice with fibrosis NaCl 0.9% treated**	0.12±0.01*	0.06±0.005*	0.18±0.01*
**Mice with fibrosis pegHYAL treated**	0.03±0.002*&	0.016±0.001&	0.05±0.02*&
**Mice with fibrosis Spiperone treated**	0.097±0.008*	0.03±0.002*&	0.128±0.011*&
**Mice with fibrosis pegHYAL and Spiperone treated**	0.070±0.004&	0.04±0.002*&	0.160±0.008*

The content of HSCs and hematopoietic progenitor cells in the lung of C57BL/6 mice on the 7th day of the experiment after single dose intratracheal BLM instillation. Membrane receptor's expression of murine HSCs derived from lung were assayed according to the protocol for BD Mouse Hematopoietic Stem and Progenitor Cell Isolation Kit (BD Biosciences, USA). The HSC population taken through a Lin^-^ selection, is shown gated Sca1^+^ and c-kit^+^, then displayed for CD34^-^ and CD34^+^. The Lin^-^ Sca1^+^c-kit^+^CD34^-^ -cells are LT-HSCs and the Lin^-^Sca1^+^c-kit^+^CD34^+^-cells are considered ST-HSCs. Thus, all two of these populations can be readily sorted from one sample. Dot plots are representative figures of three independent experiments with the mean from three independent experiments.

Results are presented as mean and SEM.

*—significance of the difference with the mice, that received intratracheal 0.9% NaCl (P <0.05),

&—significance of the difference with the mice, that received intratracheal BLM and treated with 0.9% NaCl (P<0.05), t test was used.

Thus, under a single alveolar epithelial BLM injury pegHYAL and spiperone reduce the populations of pulmonary LT-HSCs, ST-HSCs and hematopoietic progenitor cells. Administration of spiperone in 1 hour after pegHYAL increases the number of HSCs and hematopoietic progenitor cells in the lungs.

#### Mesenchymal stem cells

**Fig 5:**
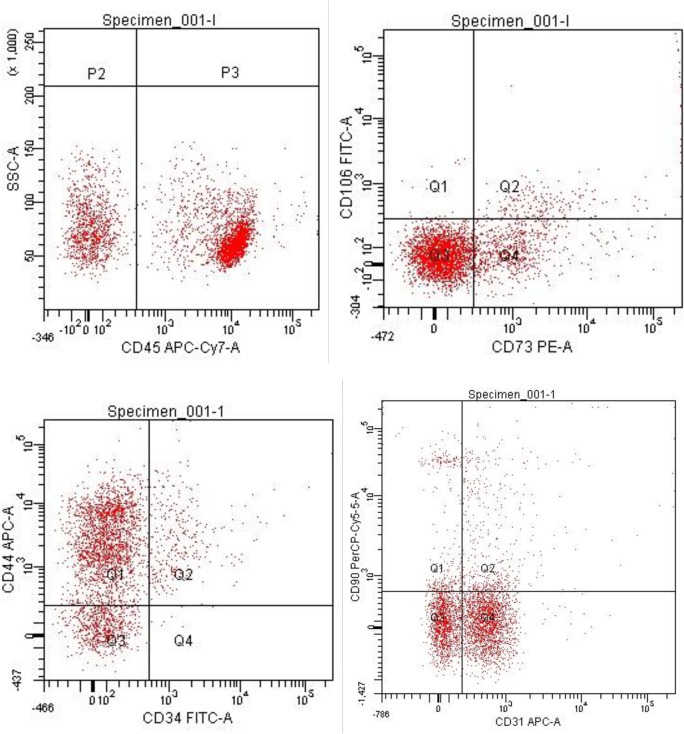
Analysis of murine pan-hematopoietic stem cells (CD45^+^-cells) and CD45^-^ -cells derived from lung (21^st^ day of the experiment). Cells were analyzed by FACS with antibody to identify mouse CD45, CD90.1, CD34, CD73, CD106 (VCAM-1), CD44 and CD31 (PECAM-1) (BD Biosciences, USA). Cells for analysis were derived from lungs of C57BL/6 mice of control and experimental groups. The CD45 negative cells population and CD45 positive cells population were sorted from one sample. The CD45 negative cells population was defined as CD34 and CD31 negative and positive for CD106, CD73, CD44, and CD90.1 at the same time in the two specimens (1 specimen—subpopulation of cells with phenotype CD45^-^, CD73^+^, CD90^+^, CD106^+^, CD44^+^ and 2 specimen—subpopulation of cells with phenotype CD45^-^, CD73^+^, CD90^+^, CD31^-^, CD34^-^). Dot plots are representative figures of three independent experiments with the mean from three independent experiments.

On the 21st day of the experiment the immunophenotype of CD45^-^ -cells was studied in accordance with the standards of mesenchymal stem cells in the lungs of C57BL/6 mice [36]. In the lungs of mice from intact control and with partially reversible pulmonary fibrosis and in mice with partially reversible pulmonary fibrosis, treated by singularly and consistently administered pegHYAL and spiperone, CD45^-^ -cells were determined expressing on the surface CD44, CD73, CD90, CD106 and were negative for CD31 and CD34 (Fig 5).

In intact control 1.7% cells of all CD45^-^ -cells from lung are MSCs (Table 5). In the fraction of lung CD45^-^ -cells in mice with partially reversible pulmonary fibrosis the desired population increased by 17.6% compared to the intact control. Influenced by pegHYAL the number of MSCs increased 2.3 times in comparison with untreated animals with pulmonary fibrosis. Spiperone reduces the MSCs population in sick animals by 30% relative to untreated pulmonary fibrosis. Administration of spiperone in 1 hour after pegHYAL administration reduces the index more effectively compared with monotherapy by pegHYAL. As it can be seen from the data presented in Table 5, the number of MSCs in the lungs of sick animals treated with two drug decreases to 1.8% of all isolated CD45^-^ -cells at 4.6% of all selected CD45^-^ -cells in the group of sick animals treated pegHYAL.

**Table 5:** Effects of pegylated hyaluronidase (pegHYAL) and Spiperone treatment on content of MSCs in the lungs derived from C57BL/6 mice with the irreversible pulmonary.

Groups	The content of MSCs
**Mice received intratracheal 0.9% NaCl**	1.7 ± 0.1
**Mice with fibrosis 0.9% NaCl treated**	2.0 ± 0.1
**Mice with fibrosis pegHYAL treated**	4.6 ± 0.2 * &
**Mice with fibrosis Spiperone treated**	1.4 ± 0.1 &
**Mice with fibrosis pegHYAL and Spiperone treated**	1.8 ± 0.1

The content of MSCs (% of all CD45^-^ -cells) in the lung of C57BL/6 mice on the 21 day of the experiment after single dose intratracheal instillation of BLM. Lung-derived MSCs were labeled with antibodies against CD45, CD44, CD73, CD90, CD106 antigens and analyzed by flow cytometry. Our data imply that on the 21st day of the experiment lung CD45^-^ -cells of mentioned above groups mice expressed on the surface of CD44, CD73, CD90, and CD106. The all of these populations is sorted from one sample. Dot plots are representative figures of three independent experiments with the mean percentage of at least three experiments.

Results are presented as mean and SEM.

*—significance of the difference with the mice, that received intratracheal 0.9% NaCl (P <0.05),

&—significance of the difference with the mice, that received intratracheal BLM and treated with 0.9% NaCl (P<0.05), t test was used.

The presented results suggest that, at partially reversible pulmonary fibrosis pegHYAL increases the amount of MSCs in the lungs of animals. Under these conditions, spiperone reduces lung MSCs population. During sequential administration of these drugs there is a significant MSCs population decrease compared with monotherapy by pegHYAL.

### Clonal activity of adherent lung mononuclear cells

In our study, a single intratracheal BLM instillation caused the generation of adherent lung mononuclear of fibroblast colonies (CFU-F) on the 21^st^ day of the experiment. The number of CFU-F with (0.50 ± 0.04)× 10^5^ adherent mononuclear cells in the intact control increased to (4.50 ± 0.25) × 10^5^ adherent mononuclear in the group with partially reversible pulmonary fibrosis (P <0.05). PegHYAL treatment has a significant (P <0.05) suppressive effect on the clonal activity of the lung adherent cells in mice with pulmonary fibrosis. In samples of lung cells of experimental group 3 the number of CFU-F is (2.50 ± 0.25) × 10^5^ of adherent mononuclear cells.

Thus, pegHYAL reduces the growth of CFU-F in the cultures of adherent lung cells in mice with partially reversible pulmonary fibrosis.

## Discussion

These investigations of pegHYAL and spiperone effects led to a series of amazing and potentially important conclusions. Firstly, we noticed that BLM induces an unusual profile of cellular migration. On the 7th day after BLM instillation hematopoietic stem / progenitor cells are registered in the lung parenchyma. The combination of surface markers of HSC, used in the present investigation, is characterized by the absence of a clone-specific markers (Lin), c-kit and / or Sca-1 (in mice) and CD34, CD133 (in man) (immunophenotype) [37, 38]. According to these combinations of markers two populations of HSCs are distinguished: long-term (Lin^-^Sca-1^+^c-kit^+^CD34^-^) and short term (Lin^-^Sca-1^+^c-kit^+^CD34^+^). HSCs are a selfrenewing and multipotent fraction of bone marrow cells, responsible for the increase of cellular blood components, including leucocytes. HSCs attraction to the lungs with fibrosis is preceded by a consecutive increase of the quantity of Lin^-^Sca-1^+^c-Kit^+^CD34^-^ -cells, Lin^-^Sca-1^+^c-Kit^+^CD34^+^-cells, Lin^-^Sca-1^+^c-kit^+^-cells (1st day) (S2 Table) and neutrophil leucocytes in bone marrow (3rd day) (S3 Table).

The main source of HSCs is the bone marrow niches. For HSCs intraosteal and vascular niches are distinguished. Osteoblasts are the main cells of the intraosteal niche [39, 40]. In the intraosteal niche HSCs are in balance with a small fraction of HSCs circulating in blood [41–43]. The vascular niche consists mainly sinusoidal endothelium cells [44]. It is possible that these cells take part in the interaction of niches [45, 46]. During bacterial and viral infections bone marrow HSCs are observed to go out of the niches [47, 48]. HSCs “lose” a lymphoid potential and are differentiated into myeloid cells [49–53]. HSCs of the animals with microbe infection are observed to migrate and accumulate in extramedullary tissues [54, 55]. Considering these results, we assumed that in BLM damage bone marrow HSCs take part in lung inflammation. BLM instillation upsets the balance between intracellular signals and the surrounding factors. In particular, the levels of chemokines and leukotrienes increase in lungs [19, 56]. This fact initiates the appearance of HSCs out of the intraosteal niche and their differentiation in myeloid direction. The indirect proof of HSCs differentiation is the increase of neutrophils in lungs [19, 28, 29]. It should be mentioned, that during the acute phase of BLM-induced inflammation macrophages and lymphocytes migrate into the lungs. The result of the inflammation secreted by the cells is seen in the increase of inflammatory cytokines in the serum and lungs (3rd day) (S1 Fig).

Cells of a mesenchymal source are one more interesting population of cells, revealed by us in the lungs. In the present work we tried to investigate the most widely distributed markers, used to characterize MSCs of mice. Pulmonary mesenchymal cells were characterized as CD31, CD34, CD45 negative and CD44, CD77, CD90, CD106 positive. We referred these cells to multipotent ones. Earlier we demonstrated the self-maintenance of these cells in a culture [28, 29]. Besides, CD31^-^, CD34^-^, CD45^-^, CD44^+^, CD73^+^, CD90^+^ and CD106^+^ cells differentiated into cells of stromal lines (adipocytes, chondrocytes, osteoblasts and fibroblasts). On the basis of cultural study and immunophenotyping we assume that the cells drawn into lungs by bleomycin (21st day—a fibrotic phase of disease) were MSC-like cells. After intratracheal instillation by BLM a population of adherent cells with high clonal activity (CFU-F) was revealed. In our opinion, the increase of CFU-F in the lungs is conditioned by a selective MSC differentiation into fibrocytes (collagen producers). Thus, MSCs contribute in the pathogenesis of IPF. In therapy the question of MSC source in lungs is of importance. MSCs are capable of migration to the injured tissues [27, 56–58]. We assume that in our investigation MSC-like cells are attracted to the fibrous lungs from bone marrow.

Secondly, in this research we showed that intranasal treatment by pegHYAL decreases a BLM-induced fibrous reaction in the lung parenchyma. The effect is seen when the preparation is prescribed in the phase of inflammation and fibrosis, in case of a single and recurrent BLM damage of alveolar epithelium. The antifibrotic activity of pegHYAL surpasses the activity of testicular HYAL. HYALs are known to be a group of enzymes that regulate HA metabolism and remodel the extracellular matrix [18]. We showed that pegHYAL decrease considerably (more than 2.8 times) the concentration of HA in case of pneumofibrosis. Testicular HYAL practically does not affect the high level of HA in sick animals (S4 Table). It is connected with a quick degradation of the unpegilated enzyme in the aggressive surroundings. According to our data the testicular HYAL is diagnosed during the first 10–15 minutes in lung homogenates of intact animals after the intranasal instillation (the data are not presented). Further its concentration decreases catastrophically. In contrast to this after the intranasal pegHYAL administration a high level of HYAL in the lungs is registered during five hours. In our opinion, the drop in speed of pegHYAL degradation is connected with polyethylene oxide, which protects the pharmacological activity of HYAL for a long time.

Thirdly, pegHYAL treatment decreases the quantity of hematopoietic stem / progenitor cells in bleomycin lungs (7-th day of inflammation). Conditioned by the parenchyma injury (BLM in particular) and inflammation [59–61] the extra quantity of HA in the extracellular matrix changes physical-chemical characteristics of the tissue [19], which leads to weakening of the extracellular matrix interaction. A space for cell migration appears [62, 63] for stem cells as well [64, 65]. Besides, interacting with proteoglycans (aggrecan, versican), HA takes part in fibrin, fibronectin and collagen organization [66]. According to our hypothesis, the break of infiltration of hematopoietic stem / progenitor cells of lung parenchyma is the result of direct pegHYAL effect on HA, destruction of profibrotic matrix of hyaluronan (space for cell migration).

The decrease in the level of TGF-β and IL-1β in the lungs (3rd day of inflammation) is an additional positive effect of pegHYAL treatment. Collagen of myofibroblasts and fibronectin of fibroblasts form the basis of extracellular matrix of fibrous lungs [67, 68]. The collagen production by fibroblasts is controlled by TGF-β [69, 70]. Interacting with CD44 receptor, HA modulates TGF-β signaling [71]. Myofibroblasts appear in fibrous lungs as a result of stromal fibroblasts differentiation. The intensity of the differentiation depends on autocrine generation of TGF-β [72]. HA serves as a necessary mediator of this mechanism [73]. IL-1β causes the proliferation of fibroblasts and chemotaxis to the site of injury [74, 75]. We assume that the decrease of BLM-induced fibrosis in the lungs after pegHYAL treatment can be connected with the reduction in production of inflammatory cytokines, caused by HA’s degradation.

By the 21st day the pegHYAL therapy of pneumofibrosis ends. During this period the increase of MSC-like cells in the lungs of treated animals is observed. A conclusion suggests itself concerning the activation of a mesenchymal constituent of fibrosis, in particular the differentiation of MSC into fibrocytes. However, during this period histopathological indices of the lungs of sick animals ameliorate. Another result of pegHYAL prescription is a drop of CFU-F fraction in lungs. Earlier C.S. Bitencourt and her colleagues (2011) demonstrated that in BLM induced pneumofibrosis the testicular HYAL enlarges the quantity of MSCs in the broncho-alveolar space. The authors explained the blockage of fibrosis and the break in collagen accumulation on the background of MSC pool expansion by decrease in the production of TGF-β [19]. It has recently been demonstrated, that MSCs can differentiate along non-stromal lineage into the epithelial lung cells (mesenchymal-epithelial transition) [76]. Similar mechanisms can be achieved by intranasal administration of pegHYAL. The pegHYAL treatment increases the number of epithelial cells in the lung (21st day after BLM instillation) (S5 Table).

Fourthly, spiperone in lungs with fibrosis reduced the concentration of collagen and hinders the deposition of fibrotic mass. In the earlier investigations it was demonstrated that lymphocytes [77–80], macrophages [81, 82] and neutrophils [83, 84] express on the surface receptors to catecholamines. On the other hand, macrophages, neutrophils [85] and T-helper cells [86–88] produce TGF-β, IL-1β and TNF-α. These results demonstrate that the decrease in the TGF-β and IL-1β levels in the serum of animals with pneumofibrosis can be caused be the inhibiting action of spiperone, conditioned by D_2_ dopamine receptors.

Receptors to catecholamines are also found on the precursors of blood cells [89–92]. In the studies by flow cytometry we found out that spiperone considerably reduces the population of MSC-like cells, LT-HSCs, ST-HSCs and progenitor hematopoietic cells in fibrous lungs. Earlier in mice with pneumofibrosis we revealed the decrease of HSCs and hematopoietic progenitor cells in bone marrow by D_2_ receptors antagonists, as well as clonal activity of bone marrow and CFU-G and CFU-GEMM circulating in the blood. Besides, spiperone had an inhibiting effect on self-maintenance and differentiation intensity of lung MSCs into the cells of stromal lineage (adipocytes, chondrocytes, osteoblasts, and fibroblast) in vitro [28, 93]. All these effects of spiperone can presumably be connected with inhibition of dopamine mechanisms of mobilization, self-maintenance, differentiation and recruiting stem / progenitor cells into the lungs tissue. We connect the disturbance in recruiting stem / progenitor cells into the lungs tissue with vascular effects of spiperone. Earlier D_1_ and D_2_ dopamine receptors in lung vessels were described [94, 95].

It should be mentioned, that spiperone blocks 5-HT_2A_/5-HT_1_ serotonin receptors and is the antagonist of α_1B_-adrenoreceptors. Presumably, by disturbing serotonin [96] and adrenergic [97, 98] mediation, the preparation inhibits the proliferation of fibroblasts and collagen synthesis.

Fifthly, pegHYAL and spiperone polytherapy inhibits the deposition of fibrotic mass more effectively than monotherapy. A high effect of polytherapy is observed when preparations are assigned during inflammatory and fibrotic phases with a single and multiple trauma of alveolar epithelium. The entire inhibition of the deposition of fibrotic mass in the lung parenchyma in polytherapy is connected by us with the inhibition of a mesenchymal component of pneumofibrosis and failure in TGF-β production.

Thus, the combination of compounds, affecting such pathogenetic elements of the forming of fibrotic matrix, dopamine mechanisms of the regulation of inflammatory cytokines production and recruiting stem / progenitor cells is potentially promising in pneumofibrosis therapy.

## Conclusions

In conditions of modelling of a single and multiple bleomycin damage of alveolar epithelium pegHYAL impedes the synthesis and deposition of collagen in the lungs of C57BL/6 mice. PegHYAL surpasses testicular hyaluronidase in activity. Spiperone enhances the antifibrotic effect of pegHYAL. Besides, pegHYAL and spiperone affect the inflammatory mediators, recruiting of hematopoietic stem / progenitor cells and MSC-like cells into fibroses lungs.

## Supporting Information

S1 Fig Effects of pegylated hyaluronidase (pegHYAL) and spiperone treatment on cytokine levels in the lung parenchyma and serum after the single dose intratracheal bleomycin instillation. At day 3rd after BLM treatment (80 μg/mouse in 0.03 ml of 0.9% NaCl) in the lungs (**A**) and serum (**B**) of C57Bl/6 mice, animals were treated with pegHYAL, spiperone and pegHYAL + spiperone (together). Interleukin (IL)-1β, tumor necrosis factor (TNF)-α and transforming growth factor beta (TGF)-β were measured in supernatant of lung tissue homogenate and serum by ELISA according to manufacturer instructions (BD Biosciences). Represent data from 2 independent experiments ± SEM. n = 5/group, *—compared to 0.9% NaCl, •—(P< 0.05) compared to bleomycin by t test.
(TIF)

S1 Table The survival rate for C57BL/6 mice after bleomycin instillation (21st day of experiment). Results are presented as percent of survival C57BL/6 mice. Over the 21 days, slightly more than half of the treated only BLM mice failed to survive the repeated BLM instillation. The HYAL, pegHYAL, Spiperone, pegHYAL and Spiperone (together) did not exhibit mortality.
(PDF)

S2 Table Number of hematopoietic stem cells and hematopoietic progenitor cells in the bone marrow of C57BL/6 mice on the 1^st^ day after bleomycin treatment. The phenotype of cells from bone marrow was studied according to the protocol for hematopoietic stem cells (BD Biosciences). The HSC population taken through a Lin^-^ selection and then Sca1^+^ and c-kit^+^ (Lin Sca-1^+^c-kit^+-^cells—hematopoietic progenitor cells), is made gated displayed for CD34^-^ and CD34^+^. The Lin^-^Sca1^+^c-kit^+^CD34^-^ cells and the Lin^-^Sca1^+^c-kit^+^CD34^+^ cells can be readily sorted from one sample. Results of three independent experiments are presented as mean and SEM. *—significance of the difference with the mice, that received intratracheal 0.9% NaCl (P <0.05).
(PDF)

S3 Table Number of metamyelocytes and neutrophilic leukocytes in bone marrow and blood of C57Bl/6 mice after bleomycin instillation. Results are presented as mean and SEM. *—compared to the mice received intratracheal 0.9% NaCl (P <0.05). Number of cells represent data from 2 independent experiments ± SEM. n = 10/group. Cell subsets were quantified by were obtained by classical hematological methods and morphologic differentiation under light microscope. Carl Zeizz Axio Lab at 100 × magnification.
(PDF)

S4 Table Effects of hyaluronidase treatment on hyaluronic acid levels. We conducted ELISA assay of HA in homogenate of right lung lobes from C57BL/6 mice at 7^th^ day after intratracheal administration of BLM. HA was determined by ELISA according to manufacturer instructions (Cusabio Biotech CO., Ltd, China). The right lung lobes were excised and snap frozen after having measured the wet weight. Sensitivities were >15.6 pg/mL. Results are presented as mean and SEM. *—compared to the mice received intratracheal 0.9% NaCl (P<0.05), &—compared to the mice received intratracheal BLM and i.n. 0.9% NaCl (P<0.05), t test was used.
(PDF)

S5 Table Effects of hyaluronidase treatment on number of epithelial cells with phenotype (CD45^-^CD31^-^CD326^+^ CD34^+^Sca-1^low^) derived from lung of C57BL/6 mice on the 21^st^ day after bleomycin instillation. Cell-surface antigens of cells derived from lung were examined by flow cytometry with the FACSCanto II flow cytometer (BD Biosciences). The data were analyzed by FACS Diva software Pro (BD Biosciences). We used the following antibodies: anti-mouse CD45 (PerCP-Cy5), CD31 (APC), CD326 (PE), CD34 (FITS), Sca-1 (PE-Cy7) (BD Biosciences). A minimum of 100,000 events were recorded for each tube. The population of epithelial cells taken through a CD45^-^ selection and then CD31^-^ and CD326^+^, is made gated displayed for CD34^+^ and Sca-1^low^. The CD45^-^CD31^-^CD326^+^ CD34^+^Sca-1^low^-cells can be readily sorted from one sample. It is shown the number of cells (% of labeled non-adherent mononuclear). Results of three independent experiments are presented as mean and SEM. *—significance of the difference with the mice, that received intratracheal 0.9% NaCl (P <0.05). &—significance of the difference with the mice with fibrosis 0.9% NaCl treated (P <0.05).
(PDF)
