# Supplementary figures and images for: Modulation of Bleomycin-Induced Lung Fibrosis by Pegylated Hyaluronidase and Dopamine Receptor Antagonist in Mice
Source: PLoS One. 2015 Apr 30;10(4):e0125065. doi: 10.1371/journal.pone.0125065 (PMC4415936; doi:10.1371/journal.pone.0125065)

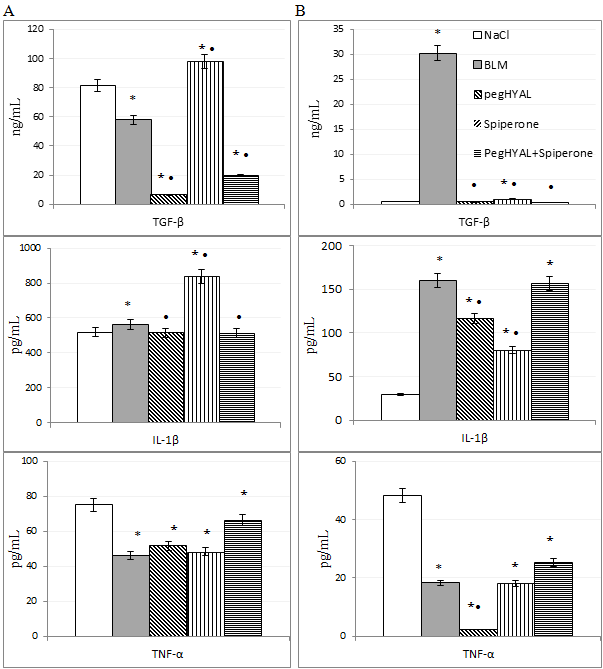

Supplement: S1 Fig — At day 3rd after BLM treatment (80 μg/mouse in 0.03 ml of 0.9% NaCl) in the lungs (A) and serum (B) of C57Bl/6 mice, animals were treated with pegHYAL, spiperone and pegHYAL + spiperone (together). Interleukin (IL)-1β, tumor necrosis factor (TNF)-α and transforming growth factor beta (TGF)-β were measured in supernatant of lung tissue homogenate and serum by ELISA according to manufacturer instructions (BD Biosciences). Represent data from 2 independent experiments ± SEM. n = 5/group, *—compared to 0.9% NaCl, •—(P< 0.05) compared to bleomycin by t test. (TIF) [file pone.0125065.s001.tif]
