# Supplementary material for: Modulation of Bleomycin-Induced Lung Fibrosis by Pegylated Hyaluronidase and Dopamine Receptor Antagonist in Mice
Source: PLoS One. 2015 Apr 30;10(4):e0125065. doi: 10.1371/journal.pone.0125065 (PMC4415936; doi:10.1371/journal.pone.0125065)
Supplement: S1 Table — Results are presented as percent of survival C57BL/6 mice. Over the 21 days, slightly more than half of the treated only BLM mice failed to survive the repeated BLM instillation. The HYAL, pegHYAL, Spiperone, pegHYAL and Spiperone (together) did not exhibit mortality. (PDF) [file pone.0125065.s002.pdf]

Table S1: The survival rate for C57BL/6 mice after bleomycin instillation (21st day of experiment)

**Groups (n – number of mice in group)**	**Percent of surviving mice**
Mice received intratracheal 0.9% NaCl (n=10)	100
**REVERSIBLE PNEUMOFIBROSIS**	
Mice with fibrosis 0.9% NaCl treated (n=10)	80
Mice with fibrosis HYAL treated (n=10)	80
Mice with fibrosis pegHYAL treated (n=10)	90
Mice with fibrosis Spiperone treated (n=10)	90
Mice with fibrosis pegHYAL and Spiperone treated (n=10)	100
**IRREVERSIBLE PNEUMOFIBROSIS**	
Mice with fibrosis 0.9% NaCl treated (n=10)	30
Mice with fibrosis HYAL treated (n=10)	60
Mice with fibrosis pegHYAL treated (n=10)	80
Mice with fibrosis Spiperone treated (n=10)	80
Mice with fibrosis pegHYAL and Spiperone treated (n=10)	90
