# Supplementary material for: Modulation of Bleomycin-Induced Lung Fibrosis by Pegylated Hyaluronidase and Dopamine Receptor Antagonist in Mice
Source: PLoS One. 2015 Apr 30;10(4):e0125065. doi: 10.1371/journal.pone.0125065 (PMC4415936; doi:10.1371/journal.pone.0125065)
Supplement: S2 Table — The phenotype of cells from bone marrow was studied according to the protocol for hematopoietic stem cells (BD Biosciences). The HSC population taken through a Lin- selection and then Sca1+ and c-kit+ (Lin Sca-1+c-kit+-cells—hematopoietic progenitor cells), is made gated displayed for CD34- and CD34+. The Lin-Sca1+c-kit+CD34- cells and the Lin-Sca1+c-kit+CD34+ cells can be readily sorted from one sample. Results of three independent experiments are presented as mean and SEM. *—significance of the difference with the mice, that received intratracheal 0.9% NaCl (P <0.05). (PDF) [file pone.0125065.s003.pdf]

Table S2: Number of hematopoietic stem cells and hematopoietic progenitor cells in the bone marrow of C57BL/6 mice on the 1st day after bleomycin treatment

$Lin^{-}Sca\text{-}1^{+}c\text{-}kit^{+}CD34^{-}$ -cells	$Lin^{-}Sca\text{-}1^{+}c\text{-}kit^{+}CD34^{+}$ -cells	$Lin^{-}Sca\text{-}1^{+}c\text{-}kit^{+}$ -cells
Mice received intratracheal 0.9% NaCl (n=8)		
0.013 ± 0.001	0.792 ± 0.082	0.809 ± 0.074
Mice with fibrosis 0.9% NaCl treated (n=8)		
0.026 ± 0.002*	0.879 ± 0.074	0.918 ± 0.091
