# Supplementary material for: Modulation of Bleomycin-Induced Lung Fibrosis by Pegylated Hyaluronidase and Dopamine Receptor Antagonist in Mice
Source: PLoS One. 2015 Apr 30;10(4):e0125065. doi: 10.1371/journal.pone.0125065 (PMC4415936; doi:10.1371/journal.pone.0125065)
Supplement: S3 Table — Results are presented as mean and SEM. *—compared to the mice received intratracheal 0.9% NaCl (P <0.05). Number of cells represent data from 2 independent experiments ± SEM. n = 10/group. Cell subsets were quantified by were obtained by classical hematological methods and morphologic differentiation under light microscope. Carl Zeizz Axio Lab at 100 × magnification. (PDF) [file pone.0125065.s004.pdf]

Table S3: **Number of metamyelocytes and neutrophilic leukocytes in bone marrow and blood of C57Bl/6 mice after bleomycin instillation**

Groups	Metamyelocytes	Neutrophilic leukocytes
**BLOOD ($\times 10^9$/ml)**		
**Mice received intratracheal 0.9% NaCl**	0.25±0.03	2.01±0.35
**Mice with fibrosis 0.9% NaCl treated**	*3rd day after bleomycin instillation*	
	0.37±0.04*	3.07±0.37*
	7th day after bleomycin instillation	
	0.38±0.04*	3.04±0.40*
**BONE MARROW ($\times 10^6$/ml)**		
**Mice received intratracheal 0.9% NaCl**	0.65±0.06	4.39±0.34
**Mice with fibrosis 0.9% NaCl treated**	*3rd day after bleomycin instillation*	
	1.36±0.19*	7.06±0.14*
	7th day after bleomycin instillation	
	1.22±0.15*	6.21±0.15
