# Supplementary material for: Modulation of Bleomycin-Induced Lung Fibrosis by Pegylated Hyaluronidase and Dopamine Receptor Antagonist in Mice
Source: PLoS One. 2015 Apr 30;10(4):e0125065. doi: 10.1371/journal.pone.0125065 (PMC4415936; doi:10.1371/journal.pone.0125065)
Supplement: S4 Table — We conducted ELISA assay of HA in homogenate of right lung lobes from C57BL/6 mice at 7th day after intratracheal administration of BLM. HA was determined by ELISA according to manufacturer instructions (Cusabio Biotech CO., Ltd, China). The right lung lobes were excised and snap frozen after having measured the wet weight. Sensitivities were >15.6 pg/mL. Results are presented as mean and SEM. *—compared to the mice received intratracheal 0.9% NaCl (P<0.05), &—compared to the mice received intratracheal BLM and i.n. 0.9% NaCl (P<0.05), t test was used. (PDF) [file pone.0125065.s005.pdf]

S4 Table. Effects of hyaluronidase treatment on hyaluronic acid levels

Groups (n – number of mice in group)	Hyaluronic acid (ng/right lung lobe)
**Mice received intratracheal 0.9% NaCl (n=5)**	66.25 ± 5.41
**Mice with fibrosis 0.9% NaCl treated (n=5)**	107.65 ± 8.52 *
**Mice with fibrosis HYAL treated (n=5)**	72.13 ± 5.23 &
**Mice with fibrosis pegHYAL treated (n=5)**	68.08 ± 4.78 &
