# Supplementary material for: Modulation of Bleomycin-Induced Lung Fibrosis by Pegylated Hyaluronidase and Dopamine Receptor Antagonist in Mice
Source: PLoS One. 2015 Apr 30;10(4):e0125065. doi: 10.1371/journal.pone.0125065 (PMC4415936; doi:10.1371/journal.pone.0125065)
Supplement: S5 Table — Cell-surface antigens of cells derived from lung were examined by flow cytometry with the FACSCanto II flow cytometer (BD Biosciences). The data were analyzed by FACS Diva software Pro (BD Biosciences). We used the following antibodies: anti-mouse CD45 (PerCP-Cy5), CD31 (APC), CD326 (PE), CD34 (FITS), Sca-1 (PE-Cy7) (BD Biosciences). A minimum of 100,000 events were recorded for each tube. The population of epithelial cells taken through a CD45- selection and then CD31- and CD326+, is made gated displayed for CD34+ and Sca-1low. The CD45-CD31-CD326+ CD34+Sca-1low-cells can be readily sorted from one sample. It is shown the number of cells (% of labeled non-adherent mononuclear). Results of three independent experiments are presented as mean and SEM. *—significance of the difference with the mice, that received intratracheal 0.9% NaCl (P <0.05). &—significance of the difference with the mice with fibrosis 0.9% NaCl treated (P <0.05). (PDF) [file pone.0125065.s006.pdf]

Table S5: Effects of pegylated hyaluronidase treatment on number of epithelial cells with phenotype ($CD45^{-}CD31^{-}CD326^{+}CD34^{+}Sca\text{-}1^{low}$) derived from lung of C57BL/6 mice on the 21st day after bleomycin instillation

Groups	Epithelial cells
**Mice received intratracheal 0.9% NaCl**	0.67 ± 0.05
**Mice with fibrosis 0.9% NaCl treated**	0.24 ± 0.02 *
**Mice with fibrosis pegHYAL treated**	0.48 ± 0.03 * &
